# Nintedanib prevented fibrosis progression and lung cancer growth in idiopathic pulmonary fibrosis

**DOI:** 10.1002/rcr2.363

**Published:** 2018-09-14

**Authors:** Kentaro Fukunaga, Shinya Yokoe, Satoru Kawashima, Yasuki Uchida, Hiroaki Nakagawa, Yasutaka Nakano

**Affiliations:** ^1^ Division of Respiratory Medicine, Department of Internal Medicine Shiga University of Medical Science Otsu Shiga Japan

**Keywords:** Idiopathic pulmonary fibrosis, nintedanib, squamous cell carcinoma

## Abstract

A 76‐year‐old man with a past history of acute exacerbation (AE) and idiopathic pulmonary fibrosis (IPF) was treated with nintedanib because of decline in his forced vital capacity over time. A new small nodular lesion was visible on a computed tomography scan of the chest before initiation of nintedanib. Disease progression in IPF and change in size of the nodular lesion were not detected during administration of nintedanib. Nine months after starting nintedanib, the patient was diagnosed with acute gangrenous appendicitis, and nintedanib treatment was discontinued. The nodular lesion increased in size four months after the cessation of nintedanib. The nodular lesion was diagnosed as squamous cell carcinoma. In this case, nintedanib inhibited the disease progression of IPF and lung cancer simultaneously. Nintedanib may play an important role in the treatment of IPF‐associated lung cancer.

## Introduction

Idiopathic pulmonary fibrosis (IPF) is a chronic and progressive fibrosing interstitial pneumonia of uncertain origin associated with a poor prognosis 1. Nintedanib is an intracellular inhibitor of tyrosine kinases that targets the fibroblast growth factor (FGF) receptor, vascular endothelial growth factor (VEGF) receptor, and platelet‐derived growth factor (PDGF) receptor 2. Randomized phase 3 trials showed that nintedanib has a number of clinical benefits in patients with IPF, such as reducing the decline of lung function and extending the time to first acute exacerbation (AE) 2. We report a case of squamous cell carcinoma in the lung associated with IPF in which tumour growth was suppressed with nintedanib therapy.

## Case Report

**Figure 1:**
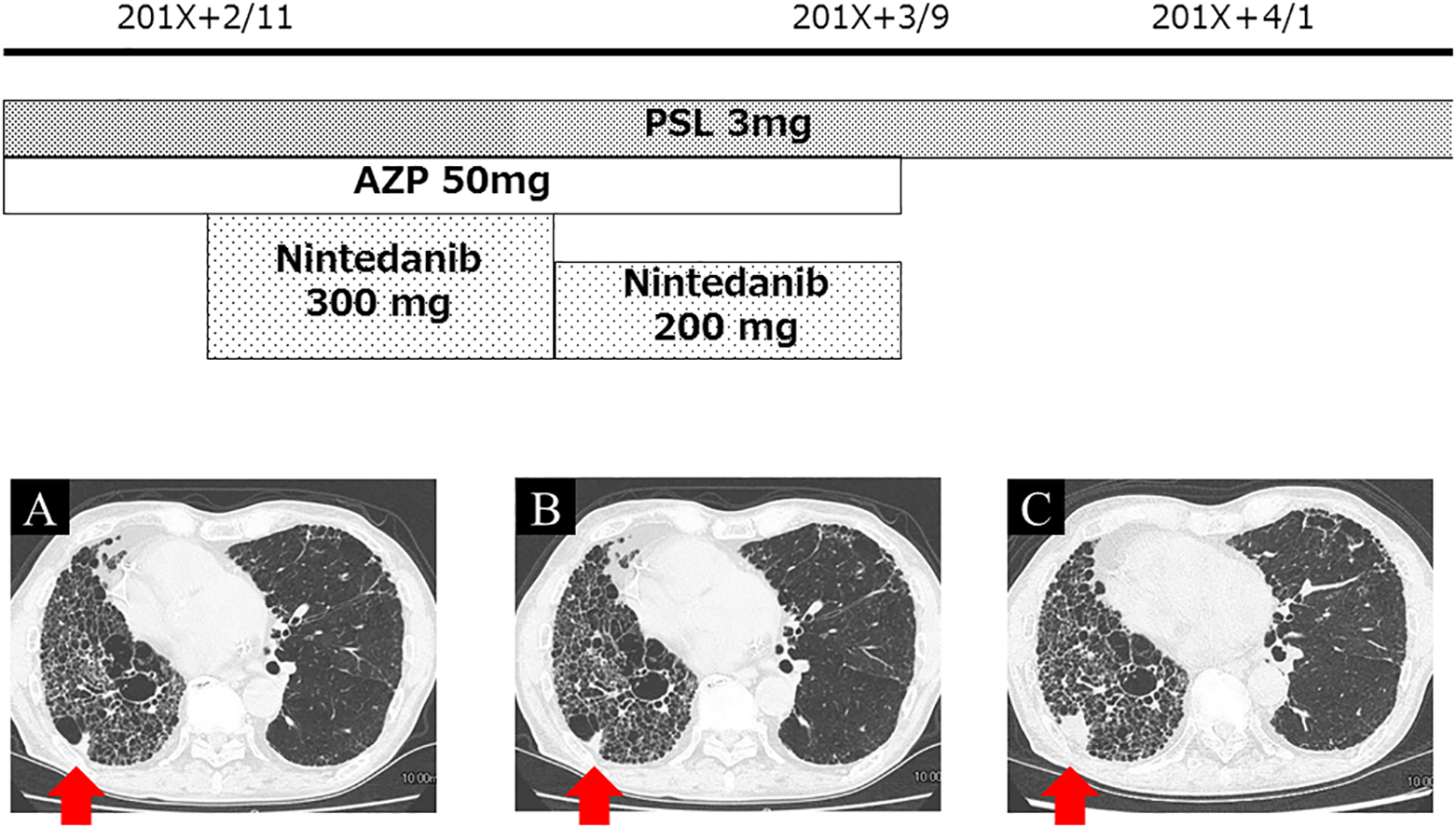
(A) Computed tomography of the chest showing a nodular lesion in the right lower lobe (arrow, 13.5 mm × 11.7 mm) at the initiation of nintedanib. (B) The nodular lesion (arrow, 12.5 mm × 10.7 mm) was almost the same size when nintedanib was discontinued because of gangrenous appendicitis. (C) The nodular lesion (arrow, 20.8 mm × 22.0 mm) was enlarged after four months of discontinuation of nintedanib.

A 76‐year‐old man, former smoker (80 pack‐years), with hypertension and hyperlipidaemia was referred to our hospital with a diagnosis of AE with IPF in 2013. After admission to our hospital, he was treated with steroid pulse therapy followed by systemic corticosteroid and cyclosporine therapy. His respiratory condition gradually improved, and corticosteroid dose was tapered. However, he needed 2 L/min of oxygen via a nasal cannula at the time of discharge. Corticosteroid dose was tapered gradually in the outpatient clinic. His forced vital capacity (FVC) declined by 8% in approximately 30 months after remission of AE. Nintedanib (300 mg/day) was administered in December 2015 because of the decline in FVC and a history of AE with IPF. A new small nodular lesion, measuring 13.5 mm × 11.7 mm (Fig. 1A), appeared adjacent to the honeycomb lung of the right lower lung lobe on a chest computed tomography (CT) scan before the initiation of nintedanib. Because of moderate deterioration of liver function after five months of nintedanib therapy, nintedanib was discontinued for two weeks and resumed after normalization of liver function at 200 mg/day.

The patient complained of right lower abdominal pain in September 2016. Acute gangrenous appendicitis was suspected on an abdominal CT scan. We observed neither deterioration of pulmonary function nor enlargement of the nodule (10.7 mm × 12.5 mm (Fig. 1B)) in the right lower lung lobe during nintedanib use. Nintedanib was discontinued, and his appendicitis improved with antibiotics.

**Figure 2:**
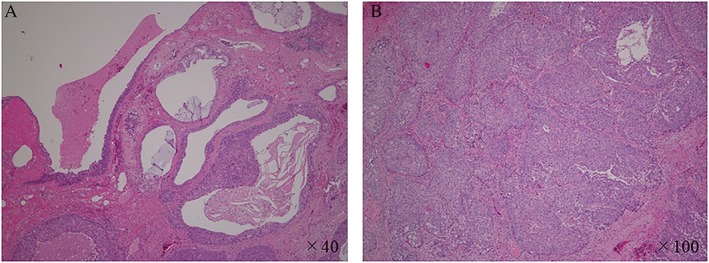
Histology of the resected nodule in the right lower lobe showing poorly differentiated squamous cell carcinoma. (A) Cancer cells developed and replaced the honeycomb lungs. (B) Polygonal cells, with an oval to irregular heterozygous nucleus, formed solid vesicular nests.

In January 2017, four months after the discontinuation of nintedanib, the nodule in the right lower lobe increased in size from 10.7 mm × 12.5 mm to 20.8 mm × 22.0 mm (Fig. 1C). The patient underwent a resection of the nodule, which was diagnosed as squamous cell carcinoma (Fig. 2).

## Discussion

IPF is considered a risk factor for the development of lung cancer 3. The incidence of lung cancer is 14 times higher in IPF patients than in the general population 4. Moreover, the cumulative incidence of lung cancer in patients with IPF increases over time 3. The prognosis of patients with lung cancer associated with IPF is significantly worse than for patients with IPF only 3. Operative intervention, radiation, and chemotherapy for lung cancer associated with IPF are difficult because they can induce AE 3.

Nintedanib inhibits tumour angiogenesis by blocking VEGF receptors 1–3, FGF receptors 1–3, and PDGF receptors α and β 5. These antitumor effects have been demonstrated in both human tumour xenograft models and patients with advanced solid tumours including lung cancer 5. The efficacy of nintedanib in advanced non‐small cell lung cancer (NSCLC) in combination with chemotherapy has been reported in the LUME‐Lung 1 and LUME‐Lung 2 trials 5. However, these trials did not evaluate the efficacy and safety of nintedanib for patients with lung cancer and interstitial pneumonia.

In our case, lung cancer was distinct and developed 30 months after initial referral to our hospital. The small nodular lesion appeared in the right lower lobe on chest CT scan before nintedanib could be started. However, the nodular lesion enlarged after cessation of nintedanib. We administered nintedanib to suppress the decline of lung function and the progression of lung fibrosis by IPF; however, nintedanib appeared to also inhibit tumour progression for nine months due to its antitumor effects.

In conclusion, we report a case of lung cancer associated with IPF in which nintedanib prevented the progression of IPF and the associated squamous cell carcinoma simultaneously because of its diverse mechanisms of action. Nintedanib may play an important role in the treatment of IPF‐associated lung cancer. However, it is still unclear whether nintedanib can reduce lung cancer incidence in patients with IPF or prolong the survival time of patients with IPF‐associated lung cancer or reduce AE associated with chemotherapy when nintedanib is administered in combination with other anticancer drugs. The Japanese Intergroup Study of Nintedanib for NSCLC with IPF (J‐SONIC) is being conducted to evaluate the safety and effectiveness of nintedanib in combination with carboplatin plus nab‐paclitaxel, and the results are eagerly awaited.

## Disclosure Statements

Y. Nakano received lecture fees from Nippon Boehringer Ingelheim Co., Ltd. (Tokyo, Japan). All authors also received research funding from Nippon Boehringer Ingelheim Co. However, the company had no control over the interpretation, writing, or publication of this work.

Appropriate written informed consent was obtained for publication of this case report and accompanying images.
